# Three cases of systemic lupus erythematosus presenting with ischemic stroke as the initial symptom: Case reports and literature review

**DOI:** 10.1002/iid3.1183

**Published:** 2024-02-08

**Authors:** Na Li, Xiaoxia Liu, Pengjia Wu, Jun Liu, Pengyu Chen, Jiashun Zeng

**Affiliations:** ^1^ Department of Rheumatology and Immunology The Affiliated Hospital of Guizhou Medical University Guiyang China; ^2^ Radiology Department The Affiliated Hospital of Guizhou Medical University Guiyang China

**Keywords:** case reports, ischemic stroke, neuropsychiatric lupus, systemic lupus erythematosus

## Abstract

**Background:**

Ischemic stroke constitutes a grave complication within the context of systemic lupus erythematosus (SLE), typically manifesting several years postdiagnosis of SLE. Incidents where ischemic stroke precedes and acts as an initial symptom of SLE are comparatively rare in its early stages, and such presentations are frequently misdiagnosed as ischemic cerebrovascular diseases, posing significant diagnostic challenges.

**Case Reports:**

This article presents three cases of young females in whom ischemic stroke emerged as the initial manifestation of SLE. It incorporates a review of 17 case reports published over the past two decades, focusing on patients with SLE where ischemic stroke was a primary symptom. This discussion encompasses the clinical presentation, outcomes, and therapeutic approaches for these patients.

**Conclusion:**

In young patients, particularly females presenting with ischemic stroke and especially in cases accompanied by hematologic or multisystemic involvement, there should be heightened vigilance for SLE‐induced ischemic stroke. Early diagnosis and treatment significantly enhance patients' quality of life and survival rates.

## Graphical abstract

Present article discusses the rarity of ischemic stroke as the initial symptom of systemic lupus erythematosus (SLE), often leading to misdiagnosis. Through case reports and literature review, it emphasizes the need to consider SLE as a diagnosis in young patients with ischemic stroke, particularly in females with associated multisystem damage, for timely treatment and improved patient outcomes.

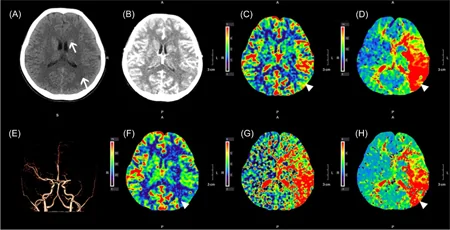

## 1 INTRODUCTION

Systemic lupus erythematosus (SLE) is a chronic autoimmune disease characterized by a loss of selftolerance, resulting in the formation of selfantigens and immune complexes that lead to multiorgan damage. Commonly affected organs include the skin, kidneys, joints, hematopoietic system, and nervous system, among others^1^ Research estimates that 5%–20%^2^ of SLE patients eventually experience neurological involvement, collectively known as neuropsychiatric SLE (NPSLE), which can manifest as a variety of neurological and psychiatric symptoms, such as headaches, cognitive impairment, and seizures. However, SLE patients who initially present with ischemic stroke as their primary symptom are relatively rare and are often prone to a misdiagnosis or underdiagnosis. In this study, we conducted a retrospective analysis of three patients treated at our center who presented with ischemic stroke as the initial symptom of SLE, along with a comprehensive analysis of 17 patients reported in 10 articles retrieved from PubMed, Web of Science, and the China National Knowledge Infrastructure database. Our aim is to enhance the understanding of this subset of SLE, where ischemic stroke is the initial presentation, and reduce the likelihood of misdiagnosis or underdiagnosis.

## 2 MEDICAL RECORD DATA

### 2.1 Case 1

**Figure 1:**
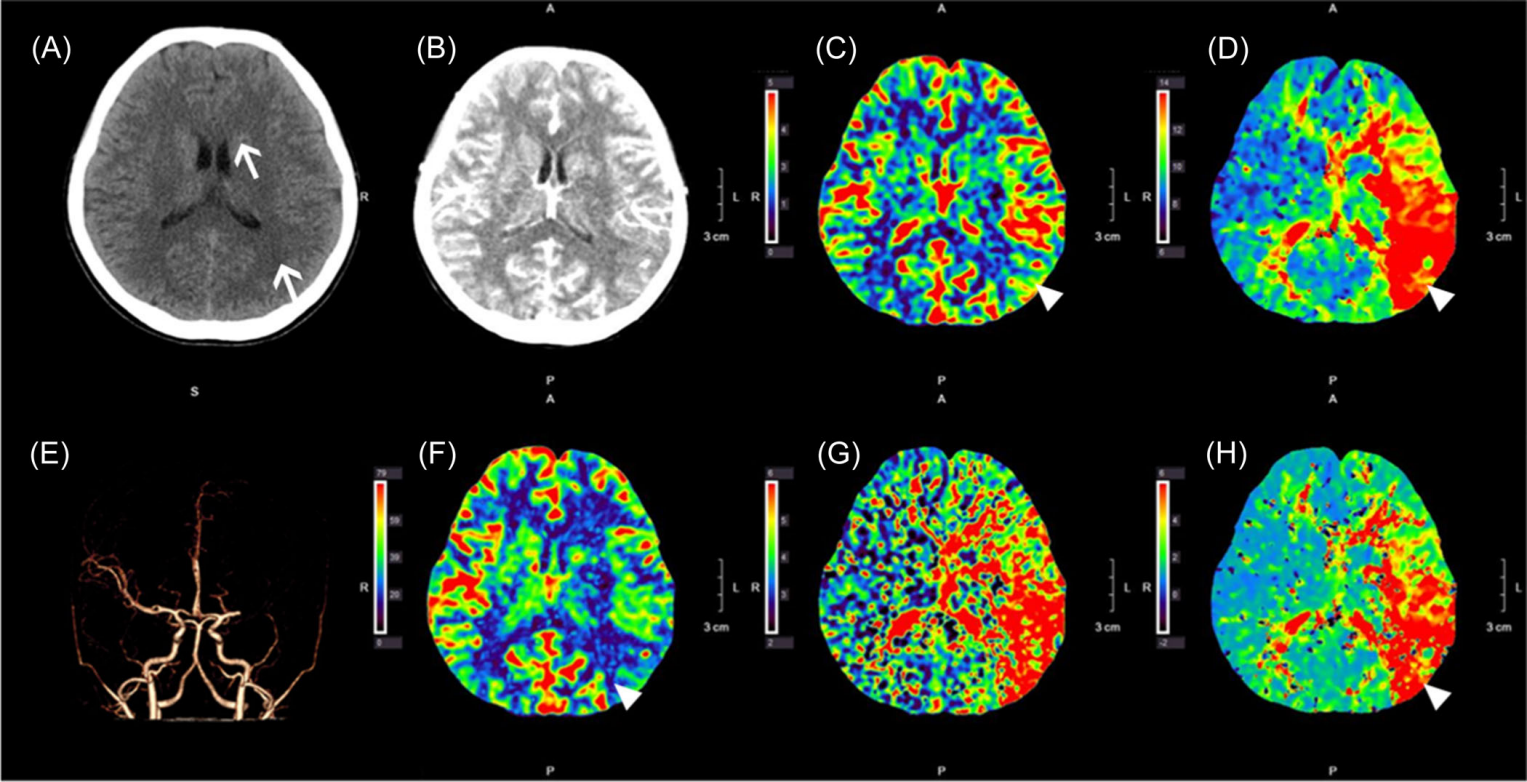
Brain imaging revealed a low‐density lesion in the left basal ganglia and parietal lobe (A, white arrowhead) with patchy, slight enhancement (B). CT perfusion imaging and CT angiography indicated a mild decrease in cerebral blood flow (CBF), prolonged time to peak (TTP), and mean transit time (MTT) in the corresponding region. The local and distal branches of the left middle cerebral artery (M1 segment) were not visualized (C–H, white triangles).

The patient, a 16‐year‐old female, was admitted to the neurology department due to the sudden onset of right‐sided limb dysfunction lasting for more than 3 h. The patient had no apparent trigger for right‐sided limb dysfunction, which manifested as an inability to raise the right upper limb and walk. An initial head CT scan at another hospital indicated a left middle cerebral artery infarction. Our stroke team conducted a head CT angiography (CTA) examination, which revealed occlusion of the left middle cerebral artery M1 segment. A head CT scan revealed a hypodense lesion in the left parietal lobe and left lentiform nucleus (Figure 1). The patient had no significant medical or psychiatric history. Upon admission, the physical examination showed the patient to be restless with mixed aphasia. Her gaze was fixed to the left, and there was a shallow right nasolabial fold. The muscle tone was reduced in the right‐sided limbs, with the muscle strength rated at 0 on the right and 5 on the left. The Babinski sign was positive on the right side and negative on the left. The patient was uncooperative for the other aspects of the physical examination The NIH Stroke Scale (NIHSS) score was 20, the Modified Rankin Scale (MRS) score was 4, and the Glasgow Coma Scale (GCS) score was 13.A chest CT scan revealed scattered solid nodules in both lungs, with patchy streaky opacities in the right lung. Laboratory tests showed a white blood cell count (WBC) of 2.34 × 10^9^/L (normal range [NR]: 4.1–11 × 10^9^/L), red blood cell count (RBC) of 3.94 × 10^12^/L (NR: 4.1–5.3 × 10^12^/L), hemoglobin (Hb) of 85 g/L (NR: 114–154 g/L), hematocrit (HCT) of 28% (NR: 36%–47%), and platelet count (PLT) of 69 × 10^9^/L (NR: 150–407 × 10^9^/L). Coagulation tests showed a prothrombin time (PT) of 15.2 s (NR: 10–15 s) and an activated partial thromboplastin time (APTT) of 87 s (NR: 28–44 s). C‐reactive protein (CRP) was 8.62 mg/L (NR: <5 mg/L), and the erythrocyte sedimentation rate (ESR) was 88 mm/h (NR: 0–26 mm/h). Urine analysis revealed proteinuria (++). Blood lipid, liver, and kidney function were within the normal ranges. Echocardiography showed no abnormalities. After ruling out absolute surgical contraindications, emergency interventional thrombectomy was performed successfully. Postoperatively, the patient received intravenous methylprednisolone to promote neural repair, hydroxychloroquine sulfate tablets, and dipyridamole to improve cerebral circulation, as well as aspirin and clopidogrel for antiplatelet therapy. Immunological tests revealed the following: antinuclear antibody (ANA) titer of 1:3200, antidouble‐stranded DNA antibodies (anti‐dsDNA): positive, anti‐SSA antibodies: strongly positive, anti‐nuclear ribosomal P protein antibodies (anti‐rRNP): weakly positive, and antihistone antibodies: strongly positive. Additionally, anticardiolipin antibodies were detected. The lupus anticoagulant initial screening test 1 (LA1) was 44 s (normal range: 31–44 s), lupus anticoagulant confirmatory test 2 (LA2): 32.10 s (normal range: 30–38 s), lupus anticoagulant standardized ratio (LA1/LA2): 1.37 (normal range: 0.8–1.2). Levels of anti‐β2‐glycoprotein I antibodies were IgM (β2‐GP1‐IgM): 23 (NR: <20 RU/mL), β2‐GP1‐IgG: 6.44 (NR: <20 RU/mL), and β2‐GP1‐IgA: 18 (NR: <20 RU/mL). The Coombs test was positive, with IgG immunoglobulin at 22 g/L (NR: 7.2–15.6 g/L) and IgM immunoglobulin at 2.74 g/L (NR: 0.5–2.55 g/L). Complement C3 was 0.727 g/L (NR: 0.7–1.4 g/L). The 24‐h urine protein (24 h UPE) was 180 mg/24 h (urine volume: 1.2 L). In accordance with the 2019 SLE classification criteria jointly released by the European Alliance of Associations for Rheumatology and the American College of Rheumatology, a definitive diagnosis of SLE, neuropsychiatric lupus, lupus hematological involvement, and antiphospholipid syndrome was made. The treatment regimen included intravenous methylprednisolone (40 mg once daily for 10 days), hydroxychloroquine sulfate tablets (two tablets twice daily), and mycophenolate mofetil tablets (three tablets twice daily) to suppress the immune response. Antiplatelet therapy with clopidogrel and aspirin was also prescribed. Reexamination of the patient after 10 days showed significant improvement in the laboratory parameters: WBC 6.32 × 10^9^/L, RBC 4.51 × 10^12^/L, Hb 100 g/L, PLT 162 × 10^9^/L, CRP 0.28 mg/L, and ESR 28 mm/h. The patient was advised to continue taking prednisone, mycophenolate mofetil tablets, and antiplatelet medications for stroke prevention and receive rehabilitation therapy upon discharge.

Reexamination 3 months later revealed the following: LA1: 40 s, LA2: 35.2 s, LA1/LA2: 1.31.

Additionally, β2‐GP1‐IgM: 32 RU/mL, β2‐GP1‐IgG: 21.3 RU/mL, and β2‐GP1‐IgA:5.91 RU/mL. During the 6‐month follow‐up, the patient continued to receive regular warfarin anticoagulation therapy and maintained a good mental status, exhibited full strength in the right limb (muscle strength grade 5), tested negative for the Babinski sign on the right side, and achieved an NIHSS score of 0. The SLE Disease Activity Index‐2000 (SLEDAI‐2000) also recorded a score of 0, indicating stable and improved clinical outcomes.

### 2.2 Case 2

**Figure 2:**
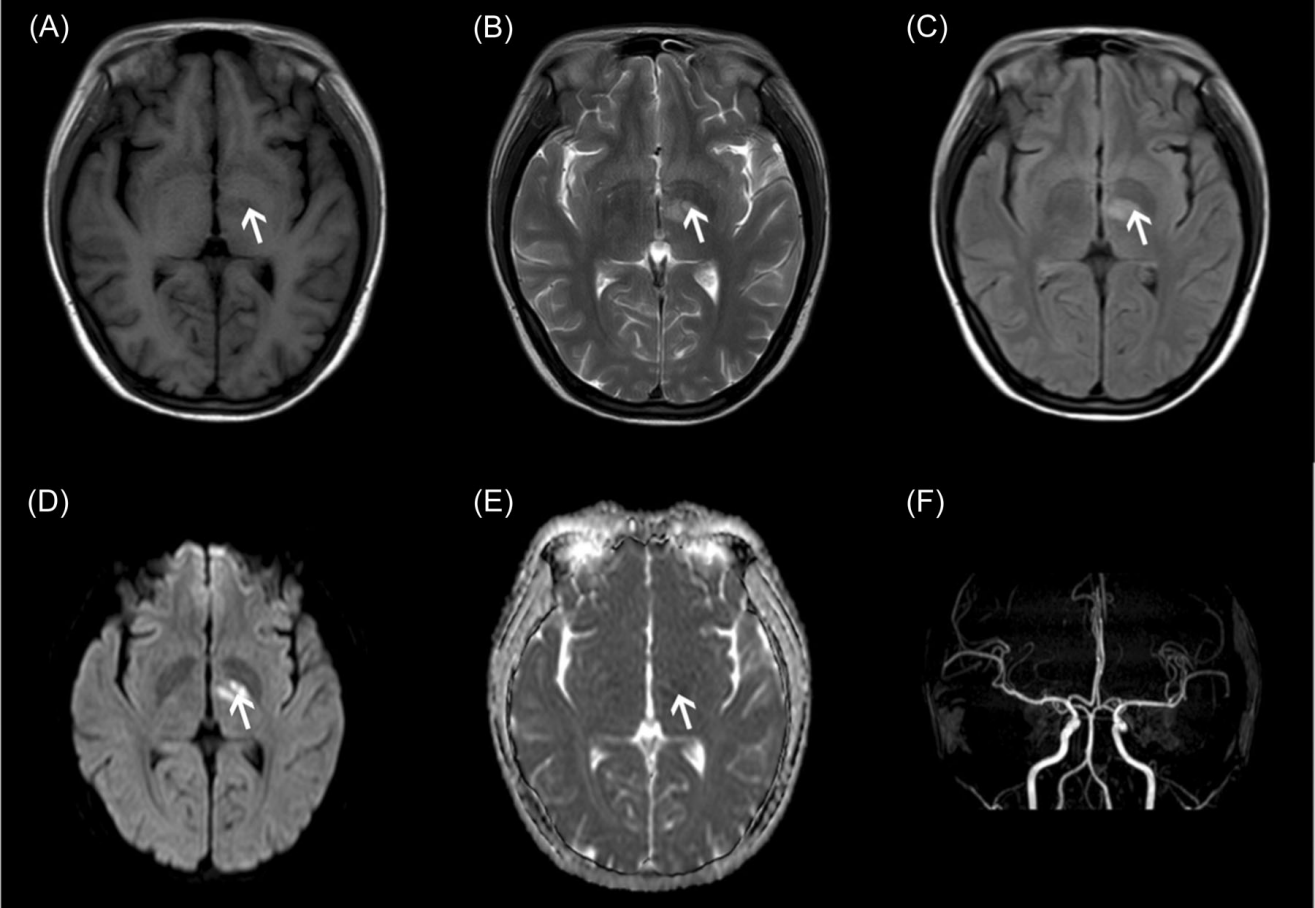
In the left basal ganglia region, small patchy areas with slightly prolonged T1 and slightly shortened T2 signal intensities are observed (A, B, white arrows). On FLAIR imaging, this area exhibits high signal intensity with blurred margins (C, white arrows). Diffusion‐weighted imaging (DWI) shows high signal intensity within this region (D, white arrows), and the corresponding ADC map shows reduced signal (E, white arrows). No abnormalities were detected on head MRA (F). ADC, apparent diffusion coefficient; MRA, magnetic resonance angiography.

The patient, a 25‐year‐old female, was admitted to the neurology department with a chief complaint of right upper limb weakness persisting for more than 2 months and sudden right lower limb weakness accompanying headache and leftward deviation of the mouth angle for 3 days. The patient denied any facial droop, drooling, or difficulty swallowing. Two days before admission, she developed dizziness and subsequently sought medical attention at the First People's Hospital of Fuquan City, where a cranial MR scan revealed “lacunar infarction in the left basal ganglia.” She received unspecified intravenous therapy without significant improvement and was further admitted to our department with a diagnosis of an “ischemic stroke.” Her personal history, obstetric history, and family history were unremarkable. Upon admission, her vital signs were stable, with a body temperature of 36.3°C, a pulse rate of 77 beats per minute, a respiratory rate of 18 breaths per minute, and a blood pressure of 112/72 mmHg. She was alert, oriented, and had clear speech. Physical examination of the heart and abdomen revealed no abnormalities. Notably, she displayed a shallow right nasolabial fold and a leftward deviation of the mouth angle. Her muscle strength was graded as 5/5 in the left upper limb, 3/5 in the distal muscles of the right upper limb, 4/5 in the proximal muscles of the right upper limb, and 4/5 in the right lower limb. Reduced sensation was noted in the right index finger. The patient demonstrated unsteady alternating movements in the right upper limb and a positive heel‐to‐shin test in the right lower limb. The left upper limb exhibited normal function, while both lower limbs displayed positive Babinski signs. The NIHSS score was 5. Laboratory tests showed the following results: WBC of 6.39 × 10^9^/L, RBC of 3.55 × 10^12^/L, Hb of 95 g/L, HCT of 30%, PLT of 267 × 10^9^/L, CRP of 5.74 mg/L, and ESR of 24 mm/h. Interleukin‐6 (IL‐6) was elevated at 62.5 pg/mL (NR: 0–7 pg/mL). Biochemical tests revealed creatinine levels at 38 µmol/L (NR: 41–73 µmol/L), albumin at 27.1 g/L (NR: 40–55 g/L), and triglycerides at 5.05 mmol/L (NR: <1.7 mmol/L). Urine analysis showed proteinuria (+). Coagulation and liver function tests were unremarkable. Echocardiography revealed mild tricuspid regurgitation, and transcranial Doppler ultrasound showed no abnormalities. Autoantibody testing revealed the following: ANA titer of 1:320, weakly positive anti‐dsDNA antibodies, strongly positive anti‐Sm antibodies, strongly positive anti‐SSA antibodies, positive anti‐rRNP antibodies, and positive anti‐histone antibodies. Additionally, perinuclear anti‐neutrophil cytoplasmic antibody (pANCA) was positive, and anti‐cardiolipin antibodies were positive. LA1: 47 s, LA2: 33.4 s, LA1/LA2:1.41. β2‐GP1‐IgM: 25 RU/mL, β2‐GP1‐IgG: 6.42 RU/mL, and β2‐GP1‐IgA: 9.92 RU/mL. The patient's immunoglobulin levels were elevated, with IgG at 25.9 g/L and IgM at 1.64 g/L. Complement 3 (C3) was decreased at 0.329 g/L. Rheumatoid factor was elevated at 36.5 IU/mL (NR: 0–20 IU/mL). The 24 h UPE was 617.6 mg/24 h (urine volume: 1.6 L). Cranial MR imaging demonstrated a nodular softening lesion with partial gliosis in the left basal ganglia region. Cranial diffusion‐weighted imaging (DWI) indicated a recent infarction in the left basal ganglia region. No abnormalities were observed in the cranial magnetic resonance angiography (Figure 2). Based on the patient's clinical presentation and diagnostic findings, in accordance with the 2019 classification criteria for SLE, a final diagnosis was established, including SLE, neuropsychiatric lupus, lupus hematological involvement, lupus nephritis, antiphospholipid syndrome, ischemic stroke, and right central facial palsy. Treatment included intravenous methylprednisolone (40 mg once daily), hydroxychloroquine sulfate tablets (two tablets twice daily), and mycophenolate mofetil tablets (two tablets twice daily). Additionally, antiplatelet therapy, anticoagulation with warfarin, and atorvastatin for lipid management were prescribed. Reevaluation after 15 days showed improved laboratory parameters, with WBC at 7.53 × 10^9^/L, RBC at 4.23 × 10^12^/L, Hb at 133 g/L, PLT at 346 × 10^9^/L, CRP at 0.31 mg/L, and ESR at 17 mm/h. A follow‐up at 3 months demonstrated complete recovery of the right‐sided muscle strength, with IgG at 15.2 g/L, IgM at 1.21 g/L, C3 at 0.717 g/L, C4 at 0.164 g/L, LA1: 43 s, LA2: 32.6 s, LA1/LA2: 1.31. β2‐GP1‐IgM: 26 RU/mL, β2‐GP1‐IgG: 26.23 RU/mL, and β2‐GP1‐IgA:4.45 RU/mL. The SLEDAI‐2000 score was 0.

### 2.3 Case 3

**Figure 3:**
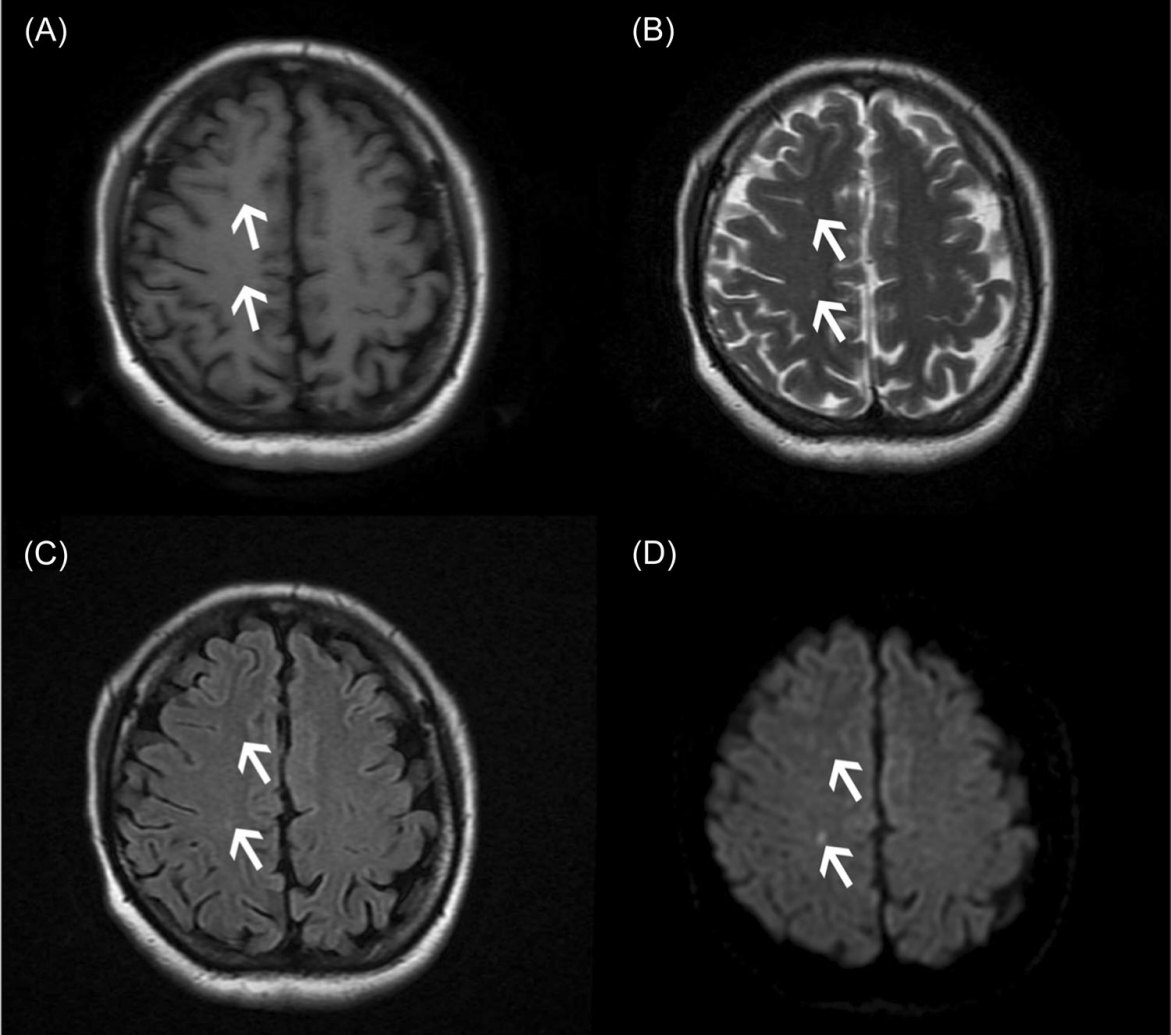
Nodular, slightly elongated T1 and T2 signal intensities are observed in the right semioval center and frontal lobe white matter (A, B, white arrows). There is slight hyperintensity on FLAIR imaging (C, white arrows), and diffusion‐weighted imaging (DWI) shows high signal intensity (D, white arrows).

The patient is a 39‐year‐old female who presented with sudden memory loss for the previous 3 days and was admitted to the neurology department. Three days before admission, the patient experienced a sudden loss of memory. She repeatedly asked her family about the current time upon waking up and could not recall specific details of events on that day. Her symptoms improved after resting, but she could still not remember the circumstances at that time and denied dizziness, headache, visual disturbances, limb numbness, difficulty swallowing, or choking while drinking. Based on the cranial MRI findings, acute cerebral infarction was suspected, and she was admitted to the hospital with a provisional diagnosis of “acute ischemic stroke.” She had a history of hypertension for the past 3 years. Upon admission, her blood pressure was 132/82 mmHg. The patient was alert, oriented, and had clear speech. She displayed intact higher cognitive functions, including calculation, comprehension, memory, and orientation. Facial examination revealed symmetric nasolabial folds, and her tongue was midline. Muscle tone and tendon reflexes in all four limbs were normal and symmetric. The muscle strength in all four limbs was graded as 5/5. Sensation to pain, temperature, and touch was normal in the limbs. Babinski signs were negative bilaterally. There were no signs of meningeal irritation. The NIHSS score was 0, and the GCS score was 15. Laboratory tests showed the following results: WBC of 4.6 × 10^9^/L, RBC of 3.98 × 10^12^/L, Hb of 107 g/L, HCT of 33.2%, PLT of 100 × 10^9^/L, PT of 14.6 s, APTT of 37 s, CRP level of 21.86 mg/L, and ESR of 53 mm/h. Urinalysis showed proteinuria (+), and serum albumin was 31.8 g/L. Blood lipid profiles, liver function, and kidney function were unremarkable. Echocardiography and transcranial Doppler ultrasound revealed no abnormalities. Carotid Doppler ultrasound showed no significant abnormalities. Cranial MR imaging revealed suspected small infarctions in the right frontal lobe and semioval center. DWI demonstrated acute infarctions in these areas (Figure 3). Perfusion‐weighted imaging (PWI) indicated no significant abnormalities in cerebral blood flow (CBF). Upon admission, the patient received aspirin for antiplatelet therapy and dipyridamole to improve circulation as symptomatic treatment. Immunological testing revealed the following results: ANA titer of 1:3200, anti‐dsDNA antibodies: positive, anti‐SSA antibodies: weakly positive, anti‐rRNP antibodies: positive, and anti‐histone antibodies: weakly positive. Additionally, pANCA was positive; anticardiolipin antibodies were negative. The Coombs test was weakly positive. The patient's immunoglobulin levels were elevated, with IgG at 20.50 g/L and IgM at 1.95 g/L. C3 was decreased at 0.28 g/L. A24h UPE was 228.6 mg/24 h (urine volume: 1.8 L). After consultation with the rheumatology and immunology department, a diagnosis was established, including SLE, neuropsychiatric lupus, and SLE with concurrent hematological involvement. Treatment consisted of intravenous methylprednisolone (40 mg once daily), hydroxychloroquine sulfate tablets (two tablets twice daily), and mycophenolate mofetil tablets (two tablets twice daily). Secondary prevention measures included aspirin, clopidogrel for antiplatelet aggregation, and atorvastatin for lipid management. The patient's condition improved significantly with treatment. Review indicator: WBC 8.47 × 10^9^/L, RBC 3.66 × 10^12^/L, Hb 116 g/L, PLT 179 × 10^9^/L, CRP 5.67 mg/L, ESR 5 mm/h. Follow‐up at 6 months showed that the patient remained alert, with clear speech and intact higher cognitive functions. The SLEDAI‐2000 score was 0.

In summary, the three patients presented with an initial diagnosis of ischemic stroke, despite the absence of skin lesions and joint pain. All three patients had hematological and renal involvement, and the final diagnosis was established through immunological investigations. The treatment approach in all three cases involved corticosteroids, immunosuppressants, and secondary prevention measures such as anticoagulation and lipid management. Notably, the patient of case 1 underwent emergent endovascular thrombectomy for acute cerebral infarction, resulting in a better prognosis than that of the other two patients. This highlights the importance of early thrombolysis treatment within the time window for acute ischemic stroke to restore blood supply to the ischemic penumbra, preserve neurological function, and improve patient outcomes.

## 3 REVIEW OF THE LITERATURE

We conducted searches in PubMed, Web of Science, and the China National Knowledge Infrastructure (CNKI) databases using the search terms “SLE” and “ischemic stroke” for literature review. The inclusion criteria for selecting articles were as follows: (1) clear diagnosis of both SLE and ischemic stroke; (2) studies involving human subjects. The exclusion criteria included: (1) duplicate data from the same center; (2) review or meta‐analyses articles. Based on this, we identified 19 studies published between 1980 and 2022 focusing on SLE associated with ischemic stroke. Among them, 9 studies were excluded as they focused on stroke occurrence post‐SLE diagnosis. Consequently, 10 articles were finalized that reported on cases of SLE with ischemic stroke as the initial presentation, documenting a total of 17 cases.^3^, ^4^, ^5^, ^6^, ^7^, ^8^, ^9^, ^10^, ^11^, ^12^ Adding the three cases presented in this study, a total of 20 cases with ischemic stroke as the initial manifestation of SLE were included. Among these cases, there were 6 males and 14 females, with ages ranging from 16 to 55 years. Eight patients showed gradual improvement after treatment, while three patients experienced severe outcomes due to delayed diagnosis and ultimately succumbed (Table 1).

**Table 1:** Clinical data of patients with systemic lupus erythematosus (SLE) with CI as the first manifestation.

Item	Case 1^3^	Case 2^3^	Case 3^3^	Case 4^3^	Case 5^4^	Case 6^5^	Case 7^5^	Case 8^6^	Case 9^7^	Case 10^7^
Age (years)	29	32	41	46	37	26	16	46	36	45
Sex	Male	Female	Male	Female	Female	Female	Female	Male	Female	Male
Clinical symptoms at onset	Quadriplegia, inability to move jaw or speak	Transient left hemiparesis immediately after delivery	Right hemiparesis three times in three years	Epilepsy, right hemiparesis, bilateral retinal obstruction	Left limb immobility and contralateral limb weakness	Headache, dizziness and slurred speech	Dizziness, headache, dysarthria, ataxia	Left limb numbness and weakness	Left limb hemiparesis	Right‐sided weakness with fluency disorder
Fever	No	No	No	No	No	No	No	Yes	No	No
Urine protein	/	/	/	/	(+)	(++)	(+)	(++)	(++)	(++)
24‐h urine protein (mg/24 h)	/	/	/	/	/	940	560	4550	/	/
IgG (g/L)	/	/	↑	/	18.49	/	/	/	/	/
Complement C3 (g/L)	↓	↓	/	↓	/	/	/	0.27	/	/
ANA titer	(+)	1:80	/	(+)	(−)	(+)	(+)	1:320	1:32	/
Anti‐dsDNA antibody	/	(+)	(+)	/	/	(+)	(+)	(+)	(+)	(+)
Anti‐cardiolipin antibody	/	/	/	/	/	(−)	(−)	(+)	/	/
Treatment	Unknown	Hormone + symptomatic support	Unknown	Unknown	No treatment	Hormone + symptomatic support	Hormone + symptomatic support	Hormonal pulse + symptomatic support	Hormone + symptomatic support	Hormone + symptomatic support
Follow‐up	/	Improved	/	/	Death	Improved	Improved	Improved	Improved	Improved

Item	Case 11^8^	Case 12^9^	Case 13^10^	Case 14^11^	Case 15^11^	Case 16^11^	Case 17^12^	Case 18	Case 19	Case 20
Age (years)	52	42	55	24	34	37	37	16	25	39
Sex	Female	Female	Female	Female	Male	Female	Male	Female	Female	Female
Clinical symptoms at onset	Left limb hemiparesis	Left limb weakness and blurred vision in the right eye	Bilateral lower limb weakness with bilateral lower limb pain	Headache, right limb weakness, and ataxia	Headache, dizziness, right‐sided and gait ataxia, paresthesia	Acute vertigo and ataxia	Acute dizziness, left‐sided and gait ataxia	Sudden right limb movement disorder	Right limb weakness	Sudden memory loss
Fever	Yes	Yes	Yes	No	No	No	No	No	No	No
Urine protein	(++)	/	(+++)	(−)	/	(−)	/	(++)	(+)	(+)
24‐h urine protein (mg/24 h)	/	/	3990	/	/	/	/	180	617	228.6
IgG (g/L)	19.4	/	16.6	/	/	/	/	22	25.9	20.5
Complement C3 (g/L)	0.45	/	0.27	↓	↓	↓	/	0.727	0.329	0.277
ANA titer	1:640	1:3200	1:3200	(+−)	(+)	(+)	(+)	1:3200	1:320	1:3200
Anti‐dsDNA antibody	(+)	(+)	(+)	(+)	(+)	(+)	(+)	(+)	(+)	(+)
Anti‐cardiolipin antibody	(−)	(+)	(+)	(−)	/	(+)	(−)	(+)	(+)	(−)
Treatment	Hormone + symptomatic support	No treatment	Hormonal pulse + symptomatic support	Heparin first, and unknown later	Unknown	Unknown	Hormonal pulse + symptomatic support	Hormone + symptomatic support	Hormone + symptomatic support	Hormone + symptomatic support
Follow‐up	Improved	Death	Death	/	/	/	Improved	Improved	Improved	Improved

*Note*: (+), positive; (−), negative; /, information not available.

Among the 20 included cases, only six were male, with the remaining 70% being female. The onset age ranged from 16 to 55 years, with an average age of 35 years. The clinical presentations of these patients with SLE at the onset of ischemic stroke were diverse and included hemiplegia (16/20, 80%), dizziness and headache (5/20, 25%), cognitive impairment (1/20, 5%), speech disturbances (3/20, 15%), dysarthria (1/20, 5%), and ataxia (5/20, 25%). Four patients experienced fever as an initial symptom, with body temperatures reaching 39°C. The remaining patients did not exhibit fever throughout their disease course. All patients did not have arthralgia during the course of the disease. Laboratory investigations revealed positive findings, including positive ANA titers (17/18, 94.4%), positive dsDNA antibodies (17/17, 100%), positive anti‐ribosomal P protein antibodies (5/7, 71.4%), positive anti‐cardiolipin antibodies (6/12, 50%), reduced C3 levels (12/12, 100%), and elevated IgG levels (6/7, 85.7%). Urinalysis indicated positive qualitative results for protein in 11 patients, with 7 of them having a 24 h UPE exceeding 150 mg. The treatment approaches were reported for only 12 patients, with nine receiving glucocorticoid therapy, three undergoing pulse steroid therapy, eight receiving anticoagulation therapy, four undergoing antiplatelet therapy, and three receiving mycophenolate mofetil treatment. Additionally, one patient each received cyclophosphamide and thrombolytic therapy. Among the 17 patients for whom outcomes were reported, three patients succumbed to the disease. One patient's death resulted from a failure to recognize the immunological mechanism early on, leading to a lack of timely steroid and immunosuppressive therapy, resulting in a gradual worsening of the disease. Another patient's death was attributed to multiple intracerebral infarctions with rapid disease progression, ultimately leading to multiorgan failure. The third patient's refusal to undergo further diagnostic workup at an early stage resulted in disease progression involving the digestive and hematological systems, leading to the family's decision to discontinue treatment.

## 4 DISCUSSION

SLE is a systemic autoimmune disease characterized by multiple organ involvement and multiple autoantibody positivity, mostly occurring in people aged 15–45 years, with an average of 29 years and a male‐to‐female ratio of approximately 1:10.^13^ The clinical manifestations of SLE are diverse, with the disease capable of initiating in any system. While mild cases may involve skin, mucous membranes, bones, and joints, severe cases can lead to neurological complications, including ischemic stroke. Ischemic stroke, a common cerebrovascular disorder, accounts for approximately 70%–80% of all cerebrovascular diseases.^14^ In 2019, the incidence of ischemic stroke in China was reported as 1700 per 100,000 population.^15^ Studies have shown that SLE patients have a 3%–20% chance of developing ischemic stroke, often within the first 5 years after diagnosis.^12^, ^16^, ^17^ However, ischemic stroke as the initial symptom of SLE is not commonly observed in clinical practice, as patients may not exhibit joint pain or a skin rash, potentially leading to a misdiagnosis or a delayed diagnosis. Currently, the exact incidence rate of this phenomenon remains unclear.

The clinical manifestations of NPSLE are diverse and can include symptoms such as headache, mood disorders, cognitive impairment, seizures, cerebrovascular diseases, psychosis, aseptic meningitis, and motor disturbances.^18^ A study based on the Chinese population identified headache, seizures, and cerebrovascular diseases as the most common types of NPSLE, with the majority of patients also showing renal and hematological involvement.^19^ Patients presenting primarily with symptoms such as headache and hemiplegia are often initially misdiagnosed with cerebrovascular diseases. Currently, there is no gold standard for diagnosing NPSLE. Different imaging techniques and the presence of autoantibodies can aid in the diagnosis of NPSLE. A meta‐analysis identified several important risk factors for NPSLE, including leukopenia, thrombocytopenia, elevated alanine aminotransferase and aspartate aminotransferase levels, fever, vasculitic skin lesions, elevated ESR, low C3 and C4 levels, and positive antiphospholipid antibodies (APL).^20^ Therefore, clinicians should remain vigilant when encountering SLE patients with these abnormal indicators.

The patients of the 20 cases presented in this study all had ischemic stroke as their initial symptom, and further laboratory investigations often revealed concomitant damage to the kidneys and hematological system. The presence of various autoimmune antibodies, such as ANA, dsDNA antibodies, and anti‐phospholipid antibodies, contributed to the diagnosis of SLE. The primary etiology of ischemic stroke is vascular wall pathology, primarily due to atherosclerosis leading to cerebral ischemia, resulting in irreversible damage to neurons in the infarcted area.^21^ Stroke is typically more common in middle‐aged and elderly individuals, with higher incidence rates in males, often accompanied by risk factors such as hypertension, hyperlipidemia, diabetes, age, and smoking.^22^ In contrast to ischemic strokes in non‐SLE patients, the patients in our study were younger, had an average age of 35 years, were predominantly female, and had almost no primary cerebrovascular disease risk factors, such as hypertension, diabetes, or hyperlipidemia. Moreover, these patients did not exhibit joint pain or a skin rash. However, laboratory tests revealed hematological system involvement in 30% of patients and renal system involvement in 84% of patients.

Ischemic stroke can be categorized into five subtypes using the TOAST classification system: (1) large‐artery atherosclerosis, (2) cardioembolism, (3) small‐vessel occlusion, (4) stroke of other determined etiologies, and (5) stroke of undetermined etiology.^23^ Studies on the most common ischemic stroke subtypes in SLE patients have yielded differing results. While some researchers have found that large‐artery atherosclerosis is the most common subtype in SLE patients,^24^ others have suggested that the most common subtype is stroke of undetermined etiology, indicating that the impact of SLE on stroke varies by subtype.^25^ Currently, only a few studies have assessed stroke subtypes in SLE patients. In our study, most patients developed ischemic stroke during the active phase of SLE, with six patients testing positive for APL antibodies. As such, the pathogenesis of ischemic stroke may be related to the following mechanisms: (1) APL antibodies induce hypercoagulability and promote thrombus formation, playing a crucial role in accelerating atherosclerosis and thrombosis formation in both arteries and veins, particularly in occlusive vascular lesions.^26^ (2) SLE patients have circulating immune complexes, complement activation, and anti‐endothelial cell antibodies. When platelet activation exposes its surface, complement activation is initiated, leading to complement cascade activation and the generation of pro‐inflammatory split products C3a and C5a. This, in turn, stimulates platelets, monocytes, and endothelial activation, resulting in vascular endothelial damage, local microthrombus formation, and fibrin deposition, ultimately causing arterial stenosis or occlusion and leading to stroke.^27^ (3) The complex interaction between the vascular endothelium, intravascular immune complexes, and other inflammatory mediators leads to central nervous system vasculitis, causing narrowing or obstruction of blood vessels and resulting in severe ischemia and necrosis. Central nervous system (CNS) vasculitis is rare and occurs in less than 1% of SLE patients.^28^, ^29^ Currently, the diagnosis of central nervous system vasculitis remains challenging, with brain tissue biopsy serving as the gold standard. In conclusion, for young female patients presenting with ischemic stroke and no prior underlying diseases, it is crucial to consider the possibility of autoimmune diseases, especially SLE‐induced ischemic stroke.

For patients with a confirmed diagnosis of NPSLE, current treatment options include glucocorticoids (GCs), immunosuppressants, biologics, intravenous immunoglobulins (IVIg), plasma exchange, and anticoagulant therapy.^30^, ^31^, ^32^, ^33^ Among the 20 cases reported in our study, only 12 reported treatment regimens. In these reports, GCs were almost universally used, but only five patients received combination therapy with immunosuppressants. This raises the question of whether SLE patients with concurrent ischemic stroke require immunosuppressive therapy. The pathogenic mechanisms of NPSLE are generally attributed to two main categories: inflammation‐mediated and ischemic injury‐induced.^34^ However, in certain circumstances, it can be challenging to differentiate between these two mechanisms, or both mechanisms may be concurrently at play. Research has shown that more than half of stroke events in SLE patients occur during periods of extensive disease activity.^35^, ^36^ SLE patients with cognitive impairment appear to benefit more from immunosuppressive therapy.^37^ Similarly, for individuals testing positive for APL, it is advisable to consider low‐dose immunosuppressive therapy. Consequently, while neuroimaging studies rarely directly observe cerebral vasculitis, the potential impact of endothelial inflammation on cerebral vasculature cannot be dismissed. Therefore, the prompt use of immunosuppressants is recommended regardless of whether ischemic stroke is the initial symptom or occurs during disease progression. In addition to the aforementioned treatments, antiplatelet and anticoagulation therapy play crucial roles in managing patients with concurrent stroke. For patients with APL‐positive SLE, independent of whether they have a history of NPSLE, antiplatelet therapy can be considered a primary prevention means, and anticoagulation treatment with warfarin should also be considered for those who meet the criteria for thrombotic APS.^38^ The use of new oral anticoagulants should usually be avoided in patients with APS due to a high risk of recurrence of arterial thrombosis.^39^ For patients with APL‐negative CI, low‐dose aspirin and other preventive measures (lipid‐lowering agents) rather than anticoagulation therapy should be used. Overall, early diagnosis and prompt and effective treatment can greatly improve the patient survival rate.

## 5 CONCLUSION

Ischemic stroke was the first manifestation of SLE in all 20 patients included in the review but was overlooked or misdiagnosed as non‐SLE‐induced cerebrovascular disease. Obviously, CNS manifestations in patients who have been diagnosed with SLE will garner the attention of clinicians, who can then determine whether such patients have NPSLE. In contrast, SLE patients with stroke as the first manifestation but no relevant clinical manifestations (e.g., arthralgia and rash) are prone to a missed diagnosis and misdiagnosis. Therefore, for ischemic stroke patients with an unknown etiology, especially young female individuals, and particularly if they present with concurrent hematological or multisystem involvement, vigilance should remain high for the possibility of stroke caused by SLE, even in the absence of rash and joint pain symptoms.

## AUTHOR CONTRIBUTIONS

**Na Li**: Conceptualization; data curation; formal analysis; investigation; methodology; visualization; writing—original draft. **Xiaoxia Liu**: Investigation. **Pengjia Wu**: Investigation. **Jun Liu**: Investigation. **Pengyu Chen**: Visualization. **Jiashun Zeng**: Conceptualization; funding acquisition.

## CONFLICT OF INTEREST STATEMENT

The authors declare no conflict of interest.

## Data Availability

The data that support the findings of this study are available from the corresponding author upon reasonable request.
